# Autophagic flux determines cell death and survival in response to Apo2L/TRAIL (dulanermin)

**DOI:** 10.1186/1476-4598-13-70

**Published:** 2014-03-23

**Authors:** Kamini Singh, Arishya Sharma, Maria C Mir, Judith A Drazba, Warren D Heston, Cristina Magi-Galluzzi, Donna Hansel, Brian P Rubin, Eric A Klein, Alexandru Almasan

**Affiliations:** 1 Department of Cancer Biology, Lerner Research Institute, Cleveland Clinic, Cleveland, OH 44195, USA; 2 Department of Anatomic Pathology, Taussig Cancer Institute, Cleveland Clinic, Cleveland, OH 44195, USA; 3 Imaging Core, Lerner Research Institute, Cleveland Clinic, Cleveland, OH 44195, USA; 4 Glickman Urological and Kidney Institute, Cleveland Clinic, Cleveland, OH 44195, USA; 5 Department of Molecular Genetics, Lerner Research Institute, Cleveland Clinic Cleveland, Cleveland, OH 44195, USA; 6 Department of Biological, Geological, and Environmental Science, Cleveland State University, Cleveland, OH, USA; 7 Department of Radiation Oncology, Taussig Cancer Institute, Cleveland Clinic, 9500 Euclid Avenue, Cleveland, OH 44195, USA; 8 Current address: Memorial Sloan-Kettering Cancer Center, 417 E68th Street, New York, NY 10065, USA; 9 Current address, Department of Pathology, University of California, San Diego, 9500 Gilman Drive, La Jolla, CA 92093, USA

**Keywords:** Autophagy, p62/SQSTM1, Caspase-8, Prostate cancer, Apo2L/TRAIL, Dulanermin

## Abstract

**Background:**

Macroautophagy is a catabolic process that can mediate cell death or survival. Apo2 ligand (Apo2L)/tumor necrosis factor-related apoptosis-inducing ligand (TRAIL) treatment (TR) is known to induce autophagy. Here we investigated whether SQSTM1/p62 (p62) overexpression, as a marker of autophagic flux, was related to aggressiveness of human prostate cancer (PCa) and whether autophagy regulated the treatment response in sensitive but not resistant PCa cell lines.

**Methods:**

Immunostaining and immunoblotting analyses of the autophagic markers p62 [in PCa tissue microarrays (TMAs) and PCa cell lines] and LC3 (in PCa cell lines), transmission electron microscopy, and GFP-mCherry-LC3 were used to study autophagy induction and flux. The effect of autophagy inhibition using pharmacologic (3-methyladenine and chloroquine) and genetic [(short hairpin (sh)-mediated knock-down of ATG7 and LAMP2) and small interfering (si)RNA-mediated BECN1 knock-down] approaches on TR-induced cell death was assessed by clonogenic survival, sub-G1 DNA content, and annexinV/PI staining by flow cytometry. Caspase-8 activation was determined by immunoblotting.

**Results:**

We found that increased cytoplasmic expression of p62 was associated with high-grade PCa, indicating that autophagy signaling might be important for survival in high-grade tumors. TR-resistant cells exhibited high autophagic flux, with more efficient clearance of p62-aggregates in four TR-resistant PCa cell lines: C4-2, LNCaP, DU145, and CWRv22.1. In contrast, autophagic flux was low in TR-sensitive PC3 cells, leading to accumulation of p62-aggregates. Pharmacologic (chloroquine or 3-methyladenine) and genetic (shATG7 or shLAMP2) inhibition of autophagy led to cell death in TR-resistant C4-2 cells. shATG7-expressing PC3 cells, were less sensitive to TR-induced cell death whereas those shLAMP2-expressing were as sensitive as shControl-expressing PC3 cells. Inhibition of autophagic flux using chloroquine prevented clearance of p62 aggregates, leading to caspase-8 activation and cell death in C4-2 cells. In PC3 cells, inhibition of autophagy induction prevented p62 accumulation and hence caspase-8 activation.

**Conclusions:**

We show that p62 overexpression correlates with advanced stage human PCa. Pharmacologic and genetic inhibition of autophagy in PCa cell lines indicate that autophagic flux can determine the cellular response to TR by regulating caspase-8 activation. Thus, combining various autophagic inhibitors may have a differential impact on TR-induced cell death.

## Background

Autophagy is a self degradation process that can mediate cell death as well as survival [1]. Autophagy induced during starvation, growth factor deprivation, hypoxia, endoplasmic reticulum (ER) stress, and microbial infection can prevent cell death [2]. However, it can be also associated with cell death due to excessive mitophagy, leading to loss of mitochondrial membrane potential (Δψ_m_), caspase activation, and lysosomal membrane permeabilization [3]. Microtubule-associated protein 1 light chain 3 beta (LC3B; also called ATG8) is used as a marker of autophagy; it is lipidated upon autophagy induction and it is required for autophagosome formation [4,5]. p62/SQSTM1 (p62) facilitates the degradation of polyubiquitinated substrates by autophagy, causing its own degradation; thus it is used as an indicator of autophagic degradation [6].

Apo2L/TRAIL (TNFSF10; clinical name, dulanermin) belongs to a small subset of pro-apoptotic protein ligands in the tumor necrosis factor (TNF) superfamily. As a soluble, zinc-coordinated homotrimeric protein, it has emerged as a promising candidate for cancer therapy through its capacity to trigger apoptosis in many types of cancers without causing significant toxicity to normal cells [7,8]. Apo2L/TRAIL has also been shown to induce autophagy-mediated cell death in glioma cells [9,10]. Induction of autophagy in PCa cells has been shown to mediate cell survival, and therefore, inhibiting autophagy enhances drug-induced cell death [11,12].

Recently, p62 has been implicated in activation of caspase-8 through Apo2L/TRAIL-mediated polyubiquitination of caspase-8 and its association with the death-inducing signaling complex [13-15]. Consistent with this notion, a study examining apoptosis in individual cells has demonstrated that differences in the extent of caspase-8 activation may be responsible for the cell-to-cell variation in Apo2L/TRAIL responsiveness [16].

In this study, we investigated the association between autophagic flux and PCa aggressiveness by immunohistochemical staining for the autophagic marker, p62, in human prostate tissue microarrays (TMA) consisting of low to high Gleason scores. In addition, we examined the effect of autophagy on cell death following Apo2L/TRAIL treatment (TR) in PCa cell lines. Our data suggest differential expression of p62 as disease progresses and that autophagic flux can determine the cellular response to TR by regulating caspase-8 activation.

## Results

### p62 is over-expressed in the cytoplasm of advanced human prostate cancer cells

To understand the role of autophagy in human PCa progression, we examined the expression of SQSTM1/p62 (p62), a marker of autophagic flux in a TMA of human prostate tissue. The TMA consisted of 51 PCa cases with Gleason Scores (GS) ranging from 6 (3 + 3) to 8 (4 + 4). Benign prostate tissue adjacent to tumor tissue was present in TMA cores from 40 of the 51 PCa cases. Using immunohistochemical staining, we determined the presence or absence of autophagy signaling by observing p62 protein localization, which, when involved in autophagic degradation, resides in the cytoplasm. In contrast, nuclear p62 participates in directing nuclear polyubiquitinated proteins to promyelocytic leukemia bodies [17], with no defined role in autophagy.

**Figure 1:**
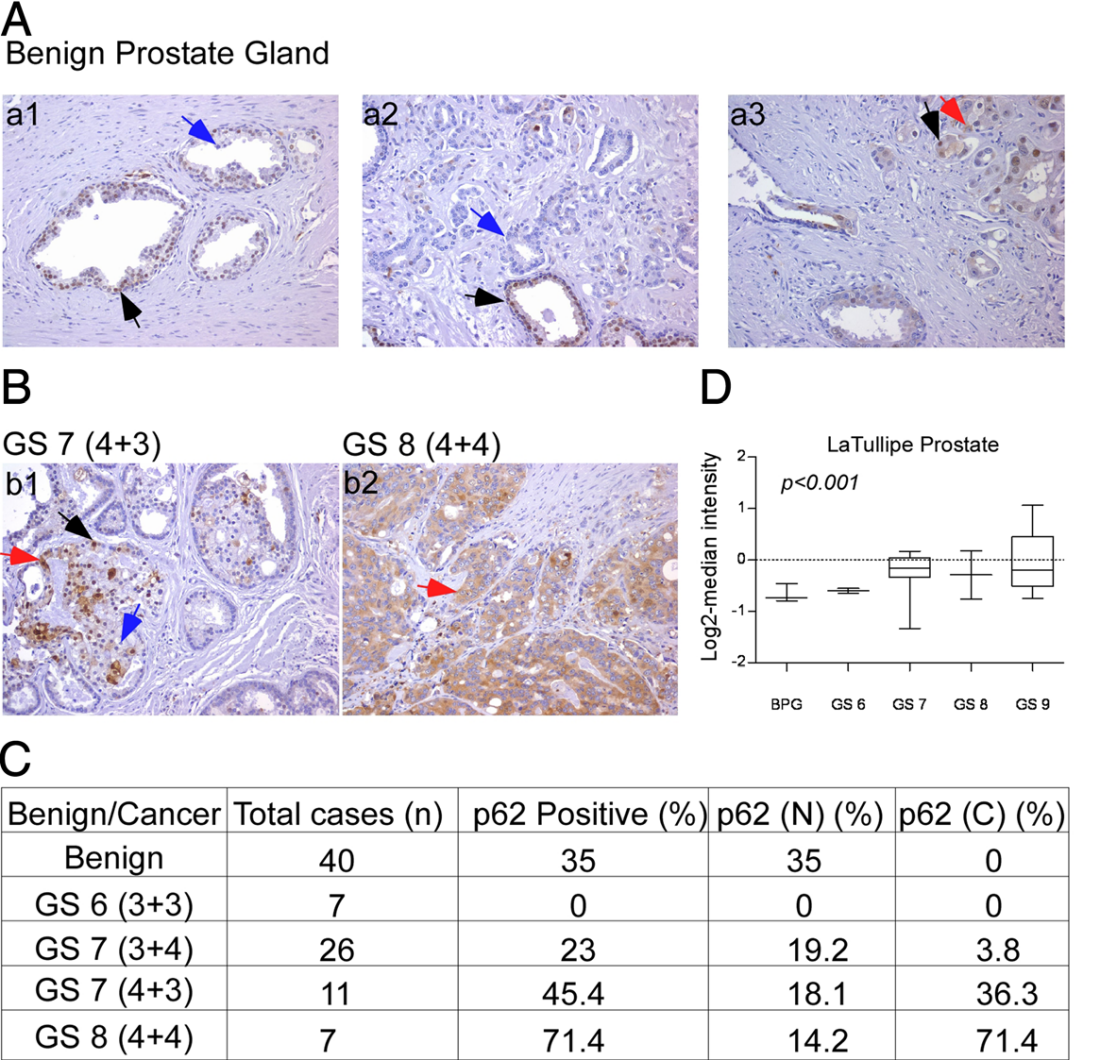
**p62 expression in human prostate cancer (PCa) samples. (A and B)** immuno-histochemical staining for p62 in PCa tissue microarrays. Black arrow: nuclear p62; red arrow: cytoplasmic; blue arrow: no staining **(a1)**, benign prostate tissue with nuclear p62 staining; **(a2)**, benign prostate tissue with nuclear p62 staining **(a3)**, benign prostate tissue displaying nuclear p62 staining and PCa with cytoplasmic staining; **(b1)**, PCa Gleason score (GS 4 + 3) 7 with both nuclear (black arrow) and cytoplasmic (red arrow) staining; **(b2)**, PCa Gleason score (4 + 4) 8 with cytoplasmic staining (red arrow). **(C)** Nuclear (N) and cytoplasmic (C) p62 expression in benign and various GS **(D)** mRNA expression analysis in benign prostate tissue (benign prostate hyperplesia) and various GS human PCa samples from the Oncomine database (p < 0.001).

Benign prostate tissue showed focal nuclear staining in 14 (35%) cases, with no evidence of cytoplasmic staining (Figure 1A and C). Remarkably, no cytoplasmic or nuclear staining for p62 was observed in any GS (3 + 3) 6 PCa cases (n = 7). Five (19.2%) of GS (3 + 4) 7 PCa cases showed nuclear staining and 1 (3.8%) showed cytoplasmic staining for p62. Interestingly, 2 (18.1%) of the GS (4 + 3) 7 PCa cases showed nuclear staining and 4 (36%) displayed cytoplasmic p62 expression (Figure 1B and C). Although there was no difference in nuclear staining between GS (3 + 4) and GS (4 + 3) (19.2% vs.18.1%), the percentage of cases with cytoplasmic p62 expression increased from 3.8% to 36% (Figure 1B and C). Cytoplasmic staining for p62 was detected in 5 (71.4%) of GS (4 + 4) 8 PCa cases, one of which showed also focal nuclear staining. These data indicate that p62 is expressed in the cell nuclei in benign prostate tissue. In contrast, cytoplasmic p62 expression increases significantly with increasing GS (p < 0.05).

Next, p62 mRNA expression was examined in two independent PCa microarray data sets available in the Oncomine database; p62 mRNA levels were increased in PCa with high GS compared to adjacent normal prostate tissue (Figure 1D). As p62 was shown to be involved in the degradation of proteins and cellular organelles by autophagy in the cytosol [18], the increase in mRNA as well as cytoplasmic localization suggested that autophagy might be a relevant process in high-grade PCa. Therefore, we further extended this study to determine how autophagy signaling in PCa affects the response to chemotherapy by investigating the effect of autophagy signaling in our TR-sensitive versus TR–resistant PCa cell lines [19].

### Apo2L/TRAIL treatment (TR) induces autophagy

**Figure 2:**
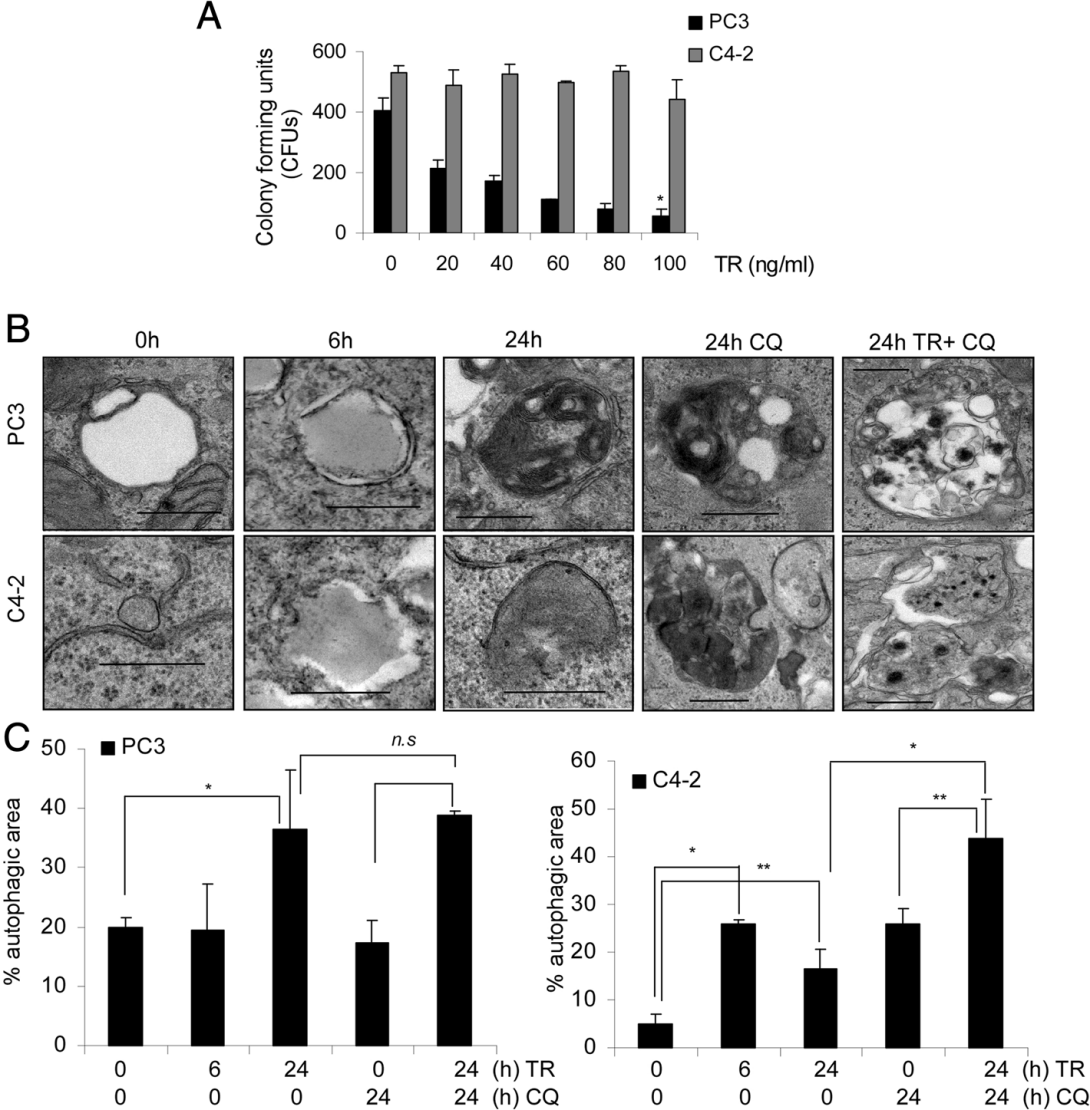
**Apo2L/TRAIL treatment (TR) induces autophagy. (A)** Clonogenic survival assay following TR at the indicated doses (*p < 0.001). **(B)** Electron microscopic analyses of autophagic structures at the indicated time following TR ± CQ. Scale bar, 500 nm **(C)** Quantitative analysis of the autophagic area normalized to the cytoplasmic area following TR ± CQ (*p < 0.001, **p < 0.05). Data represent means ± SEM (>3 different fields per sample).

LNCaP-derived C4-2 human PCa cells are resistant to TR [19,20]. Following TR, clonogenic survival declined by up to 10-fold in PC3, but not in C4-2 cells (Figure 2A). To study the role of autophagy in regulating cell survival, we examined the induction of autophagy in these cell lines. Detecting the presence of autophagic vesicles by using transmission electron microscopy (TEM) is one of the most widely used and sensitive techniques to monitor autophagy [21]. Autophagosomes are typically identified as vacuolar structures bordered by a thin membrane, sequestering cellular contents. The immature vesicles have an electron density lower than or equivalent to the cytoplasm whereas the mature or late-stage vesicles are characterized by higher density. We show such representative structures in Figure 2B. For quantification in Figure 2C, the area covered by these structures was measured and normalized to the total cytoplasmic area analyzed. TEM data suggested that more autophagosomes were present constitutively in PC3 compared to C4-2 cells (Figure 2B and C). In C4-2 cells there was an early but transient increase in the number of autophagosomes at 6 h following TR, which declined by 24 h (Figure 2B, bottom and C, *right*). In PC3, however, autophagosome formation significantly increased only after 24 h TR (Figure 2B, bottom and C, *left*). Also, TR plus chloroquine (CQ), which inhibits autophagic degradation, compared to CQ alone, resulted in a greater accumulation of autophagosomes in C4-2 compared to PC3 cells, suggesting that autophagic flux was higher in C4-2 cells (Figure 2B and C). These results demonstrate that C4-2, but not PC3 cells, exhibit a rapid induction and effective completion of autophagy in response to TR.

**Figure 3:**
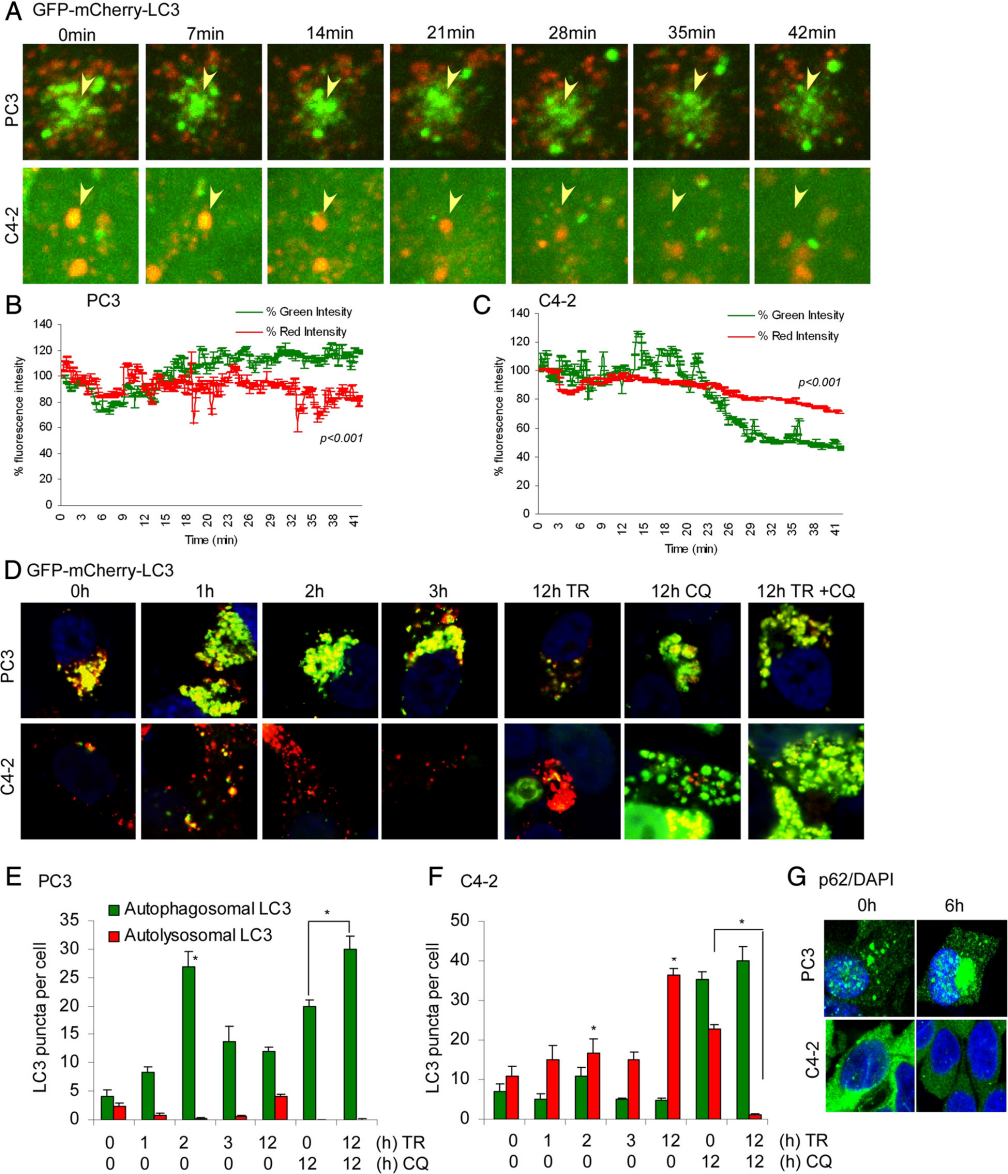
**The autophagic flux is higher in C4-2 compared to PC3 cells. (A-C)** Representative confocal time lapse images every 7 min and quantitation of green and red fluorescence for LC3B expression using GFP-mCherry-LC3 fusion protein stably expressed in PC3 and C4-2 cells following TR. **(D-F)** representative confocal images of fixed cells stably expressing GFP-mCherry-LC3 and quantitation of autophagosomal/autolysosomal LC3B-II puncta in PC3 and C4-2 cells following TR ± CQ. Yellow shows co-localization of GFP and mCherry, indicating completed autophagosomes (*p < 0.001). Data represent means ± SEM. **(G)** Representative confocal images of fixed cells for p62 (green) immunostaining following 8 h TR in PC3 and C4-2 cells. Nuclei were stained with DAPI.

Next, we confirmed autophagic flux by determining LC3B localization using GFP-mCherry-LC3, which allows distinction between autophagosomal and autolysosomal LC3B as yellow (indicating co-localization of GFP and mCherry) and red signals, respectively. By time-lapse confocal imaging, PC3 cells showed no change in green and red fluorescence for LC3B until 42 min, suggesting that autophagosomes remained intact following TR (Figure 3A and B; Additional file 1: Movie S1 and Additional file 2: Movie S2). In contrast, C4-2 cells showed decreased green fluorescence intensity for LC3B at 21 min, with a small decrease, if any, in red fluorescence, indicating that autophagosomes were converting into autolysosomes (Figure 3A and C).

Next, confocal imaging was performed to determine the total number of autophagosomes and autolysosomes, by scoring LC3B puncta following TR ± CQ. PC3 cells displayed only autophagosomal LC3B-II (yellow puncta on co-localization), whereas C4-2 showed more autolysosomal LC3B-II (red) within 2 h of TR (Figure 3D). Co-treatment with CQ led to accumulation of TR-induced autophagosomal LC3B-II to a larger extent in C4-2 compared to PC3 cells (Figure 3D). Quantitation of autophagosomal and autolysosomal LC3B puncta also revealed much higher autophagic flux in response to TR in C4-2 compared to PC3 cells (Figure 3E and F). Thus, autophagic flux observed in C4-2 cells was associated with cell survival whereas the low flux in PC3 cells was associated with TR-induced cell death.

Immunofluorescence staining for endogenous LC3B showed an increase in the number of LC3B puncta in C4-2 but not PC3 cells following 8 h TR (Additional file 3: Figure S1A). PC3 cells had, however, more LC3B puncta constitutively compared to C4-2 cells, indicating a greater autophagosomal content at baseline, but with no further induction of autophagy following TR. Addition of CQ led to accumulation of LC3B puncta in both PC3 and C4-2 (Additional file 3: Figure S1A). In PC3 cells the number of LC3 puncta did not significantly increase following TR plus CQ compared to CQ alone. However, in C4-2 cells the number significantly increased following TR and was even higher upon addition of CQ (Additional file 3: Figure S1A).

Similar results were obtained with analysis of p62 levels. An autophagy-specific substrate that acts as a scaffold protein and forms protein aggregates, p62 is directed with its targets to autophagic degradation [22,23]. As shown by confocal immunostaining (Figure 3G) and western blotting (Additional file 3: Figure S1B), after TR expression of cytoplasmic p62 aggregates are increased in PC3, whereas p62 expression decreased in C4-2 cells. Pretreatment with CQ, however, prevented TR-induced degradation of p62 in C4-2 cells (Additional file 3: Figure S1B). These findings were extended to all additional PCa cell lines examined. Similar to C4-2, LNCaP, DU145, and CWRv22.1 cells exhibited minimal cell death (< 10%) as opposed to ~ 50% cell death in PC3 cells following TR (Additional file 3: Figure S1C). Similar to C4-2 cells, p62 levels as analyzed by western blotting and immunostaining, were decreased in the other TR-resistant cell lines following TR treatment (Additional file 3: Figure S1D and S1E). Nevertheless, CQ treatment inhibited TR–induced degradation of p62 (Additional file 3: Figure S1D and S1E). These results suggest that in response to TR, autophagy is induced and high autophagic flux is associated with cell survival whereas low flux is associated with cell death.

### Inhibition of autophagy enhances TR-induced cell death in C4-2, but not PC3 cells

**Figure 4:**
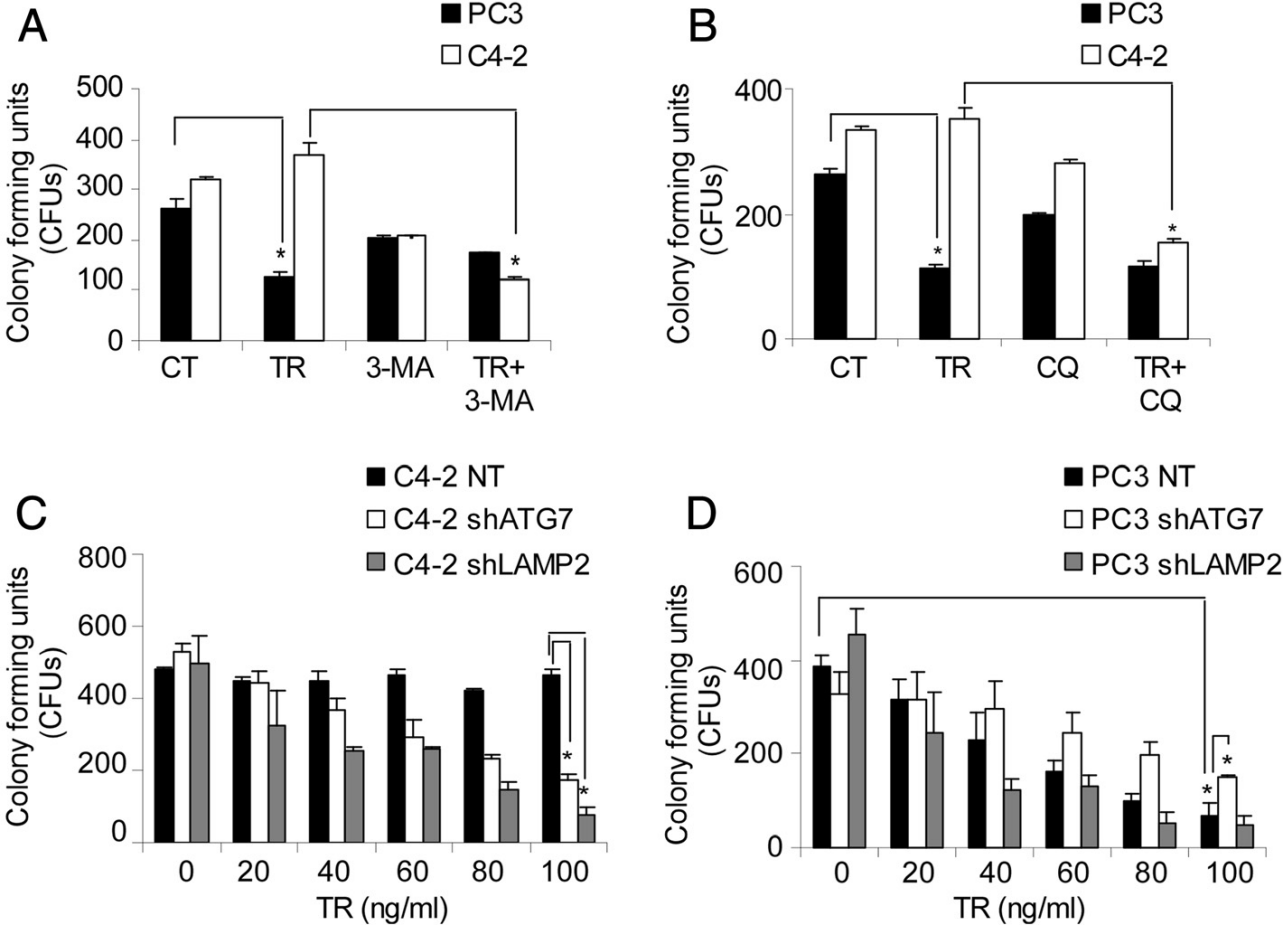
**Autophagy inhibition prevents cell death in PC3 but enhances cell death in C4-2 cells. (A)** Clonogenic survival assay in PC3 and C4-2 cells following TR (40 ng/ml) ± 3-methyladenine (3-MA) (*p < 0.001). **(B)** Clonogenic survival assay following TR ± CQ (*P < 0.001). Clonogenic survival in **(C)** C4-2 and **(D)** PC3 cells stably expressing non-target shRNA, shATG7, or shLAMP2, with the indicated doses of TR (*p < 0.001).

Next, pharmacological and genetic approaches were used to study the effect of autophagy induction and completion on TR-induced cell death in PCa cell lines. Pharmacological inhibition of autophagy by 3-MA or CQ sensitized C4-2 cells to TR-induced cell death, with no significant effect on PC3 cells (Figure 4A and B). For genetic inhibition of autophagy, lentiviral-mediated shRNA-expressing stable clones of ATG7 and LAMP2 were generated in PC3 and C4-2 cells. LC3B-II levels were reduced in shATG7, while they increased in shLAMP2-expressing cells (Additional file 4: Figure S2A and S2B). p62 levels were increased in both shATG7- and shLAMP2-expressing C4-2 and PC3 cells, suggesting that autophagy inhibition resulted in accumulation of p62 (Additional file 4: Figure S2A and S2B). The extent of knockdown was > 50% for both ATG7 and LAMP2 in PC3 and C4-2 cells (Additional file 4: Figure S2A and S2B). As with 3-MA and CQ, ATG7 and LAMP2-depleted C4-2 cells formed fewer colonies (Figure 4C) and underwent cell death (Additional file 5: Figure S3A) in response to TR, indicating that they were more sensitive to TR when autophagy was inhibited. Interestingly, ATG7 depletion in PC3 cells inhibited TR-induced cell death significantly (Figure 4D and Additional file 5: Figure S3A). Thus in TR-sensitive cells, with an intrinsic defect in TR-induced autophagic flux, inhibition of autophagosome formation at an early step was protective.

Overall, our data suggest that autophagic clearance of toxic cellular components is essential for the PCa cells to survive TR-induced cell death that is associated with autophagy induction. In TR-sensitive cells TR induces autophagosome-formation; however, due to impaired autophagic flux, autophagosome-associated toxic cellular aggregates are formed, and this results in cell death. Therefore, inhibiting autophagy induction could antagonize its effect. In TR-resistant cells that are proficient in autophagic flux, TR-induced accumulation of cellular aggregates is prevented and the cells survive. Thus, inhibition of the autophagic pathway in TR-resistant cells leads to accumulation of protein aggregates and sensitizes these cells to TR. Thus, TR-induced autophagy causes cell death in TR-sensitive cells, whereas it has a prosurvival role in TR-resistant cells due to differential autophagic flux.

**Figure 5:**
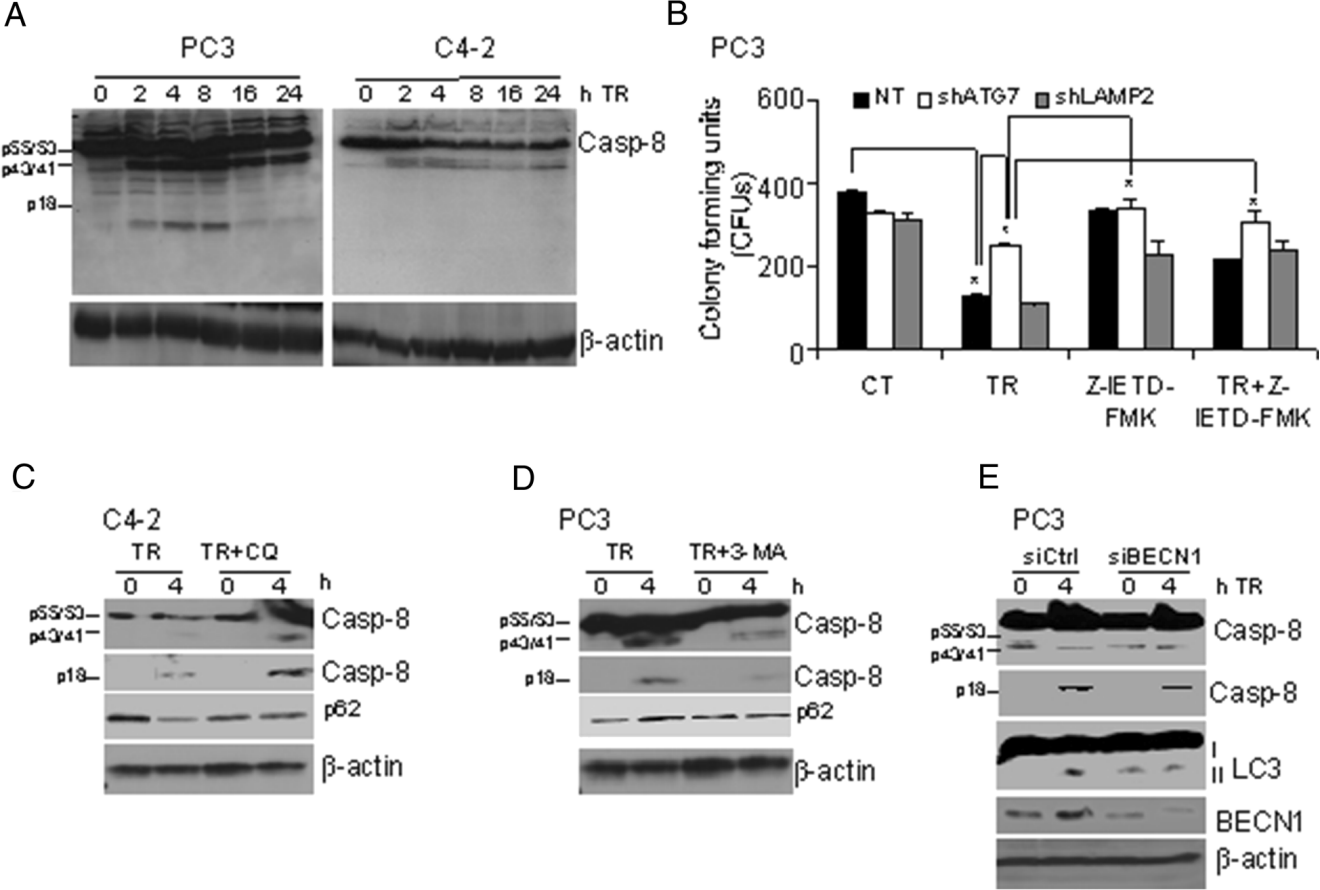
**Impaired autophagic flux causes apoptosis in PCa cells by caspase-8 activation. (A)** Western blot analysis for caspase-8 activation following TR. β-actin served as loading control. **(B)** Clonogenic survival in PC3 cells stably expressing non-target shRNA, shATG7, or shLAMP2, after TR (40 ng/ml) ± caspase-8 inhibitor z-IETD-fmk (10 μM) for 24 h (*p < 0.001). Western blot analysis of the indicated proteins in **(C)** C4-2 cells following TR ± CQ, and **(D)** PC3 cells following TR ± 3-MA or **(E)** PC3 cells expressing non-target siRNA or siBECN1 following TR.

Caspase-8 can be proteolytically cleaved to a p18-kD fragment through its association with p62 aggregates, leading to its complete activation and ensuing apoptosis [13]. Since differential autophagic flux in PCa cells determined cell death in response to TR, we investigated whether the impaired or inhibited autophagic flux led to cell death in response to TR by accumulation of p62 and subsequent activation of caspase-8. Our data suggest that, indeed, PC3 cells with impaired flux showed the pro- and cleaved (p43/p41)-forms of caspase-8 and its fully activated p18-kD form following TR (Figure 5A). In contrast, C4-2 cells showed only the p43/p41 forms of caspase-8, indicating that the full activation of caspase-8 necessary for apoptosis was absent (Figure 5A). TR-induced cell death was significantly impaired in PC3, with minimal effect on C4-2 cells following inhibition of caspase activation by the pan-caspase inhibitor z-VAD-fmk or the caspase-8 specific inhibitor z-IETD-fmk, as determined by annexinV/PI staining (Additional file 5: Figure S3B). z-IETD-fmk inhibition of caspase-8 also prevented cell death in PC3 cells expressing shATG7 and shLAMP2 (Figure 5B). Consistently, in C4-2 cells inhibition of autophagic flux using CQ pretreatment, as measured by inhibition of p62 degradation following TR treatment (Figure 5C), led to TR-induced accumulation of the fully activated p18-kD form of caspase-8 (Figure 5C). Similarly, in PC3 cells both 3-MA pretreatment and siBECN1-expression led to a decrease in TR-induced cleaved caspase-8 levels (Figure 5D and E, respectively). These results confirmed that autophagy induction was required for TR-induced apoptosis in PC3 cells, which depended on caspase-8 activation.

Thus, a constitutive defect in autophagic flux in response to TR causes inhibition of autophagic clearance of p62 aggregates that, in turn, results in caspase-8 activation, leading to cell death in PC3 cells. However, in TR-resistant C4-2 cells, complete autophagy signaling leads to clearance of p62 aggregates, and hence activation of caspase-8 is prevented, thereby facilitating cell survival.

## Discussion

In this study we show that autophagy is critical for PCa pathogenesis, as p62 is overexpressed in the cytoplasm of high grade PCa. In contrast, in benign tissue it is only expressed in the cell nuclei, suggesting that p62 has a more basic function apart from autophagy [17]. Interestingly, cytoplasmic p62 expression is positively associated with the aggressiveness of the disease. These findings suggest that p62 could be a potential molecular biomarker for PCa progression and that elevated autophagy might be an important factor for disease progression, maintenance of tumor homeostasis in higher grade PCa, or both. In addition to its principal role of maintaining cellular homeostasis in health and disease, during chemotherapy, autophagy counterbalances the cellular stress generated by chemotherapeutic agents as well as provides energy to maintain cellular homeostasis [24,25]. Therefore, autophagy inhibition has recently emerged as a potential therapeutic approach to induce cell death in cancer cells. The dependence of PCa on this pathway is, therefore, exploitable for therapeutic benefit.

We have previously shown that the combination of CPT-11 with TR increases apoptosis in C4-2 PCa cells, which are otherwise resistant to TR [19,20]. Autophagy mediates cell survival in tumor cells and serves as a mechanism of resistance against many chemotherapeutics, including TR [26]. Here, we have identified autophagy as a mediator of cell survival in four TR-resistant PCa cell lines. These TR-resistant PCa cells exhibited high autophagic flux, in contrast to TR-sensitive PC3 cells, in which autophagic flux was low, preventing completion of autophagy and leading to autophagosome accumulation.

TR led to degradation of p62 and prevented caspase-8 activation in TR-resistant cells. However, in TR-sensitive cells, accumulation of autophagosomes and p62 protein aggregates, which were associated with impaired autophagic degradation, led to caspase-8 activation and apoptosis. Consistently, inhibition of autophagy pharmacologically or genetically by shRNA-mediated knockdown of ATG7 and LAMP2 sensitized C4-2 cells to TR. Importantly, inhibition of autophagy at the different steps in the autophagy pathway led to different outcomes for TR-induced cell death in PC3 cells. Thus, when autophagy was inhibited by 3-MA, siBECN1, or shATG7 before the association of LC3 with the autophagosomal membranes, p62 aggregate formation and subsequent caspase-8 activation was prevented, and cell death was inhibited. In contrast, inhibiting autophagic degradation using CQ or shLAMP2 had no effect on TR-induced cell death in PC3 cells. These findings suggest that accumulation of autophagosomes and p62 protein aggregates in the absence of autophagic degradation is sufficient for TR-induced cell death. Levels of LAMP2 were decreased in TR-sensitive compared to TR-resistant cells (data not shown), supporting the notion that autophagic degradation was inhibited in TR-sensitive cells. This was also true in a small-lung carcinoma model, where LAMP2 was down-regulated in TR-sensitive as compared to TR-resistant groups [14]. These findings further support our conclusion that autophagic degradation is impaired in TR-sensitive tumor cell lines.

## Conclusions

In summary, we define how the extent and nature of autophagic signaling can determine the response to TR that may be exploited for further clinical development of dulanermin or agonistic antibodies against the Apo2L/TRAIL receptors. Establishing the extent of autophagy in different grades of PCa will be useful for designing better therapeutic modalities by combining autophagy inhibitors currently in clinical trials [27]. It has been recently suggested that of the NIH-funded PCa clinical trials currently recruiting, 60% are testing interventions known to exert at least a moderate effect on autophagy [28]. These include autophagy inhibitors (e.g. hydroxychloroquine) and therapeutics that impact autophagy, either directly, such as mTOR inhibitors (e.g. everolimus), or indirectly, such as inhibitors of PI3K and AKT. Moreover, in patients with caloric or metabolic deregulation (such as those who are obese) autophagy may have a greater impact, as might agents that modulate it and, therefore, based on our findings, such patients could be stratified for individualized treatments.

## Methods

### Reagents and plasmids

Recombinant Apo2L/TRAIL (dulanermin) was a generous gift from Genentech. The following reagents were purchased: chloroquine (CQ), 3-methyladenine (3-MA), acridine orange, and propidium iodide (Sigma-Aldrich). The GFP-mCherry-LC3B and pCL10 plasmids were a kind gift from Dr. Jayanta Debnath (University of California San Francisco). The lentiviral packaging plasmids pVSVG and dr 8.7 were from Invitrogen. pLKO.1–puro control vector, shATG7, and shLAMP2 (SHCLNG-NM_006395, SHCLNG-NM_002294) Mission shRNAs were from Sigma-Aldrich. siBECN1 (sc-29797) and control siRNA (Fluorescein Conjugate)-A (sc-36869) were from Santa Cruz Biotechnology. Fugene was from Roche Applied Science. The following antibodies were used; p62 (Fitzgerald Industries International); LAMP2, p62, BECN1 (Santa Cruz Biotechnologies); ATG7 (Cell Signaling Technologies), *β*-actin (Sigma Aldrich); Caspase-8 (C-15; Alexis); secondary anti-mouse HRP (Millipore); and secondary anti-rabbit HRP (Fisher Scientific).

### Cell culture and treatments

PCa cell lines PC3, LNCaP, DU145, CWRv22.1, and LNCaP-derived C4-2 were maintained in RPMI 1640 media. All media were supplemented with 10% fetal bovine serum (FBS, Atlanta Biologicals), L-glutamine, and 100 unit/ml penicillin-streptomycin (Invitrogen). Cells were grown in a humidified incubator at 37°C and 5% CO_2_. Cells were treated with 100 ng/ml Apo2L/TRAIL (dulanermin) or CQ and 10 mM of 3-MA, unless specified otherwise.

### Tissue specimens

Human prostate tissue was from 51 radical prostatectomy specimens from patients with PCa with different Gleason score (GS) including GS (3 + 3) 6 (n = 7), GS (3 + 4) 7 (n = 26), GS (4 + 3) 7 (n = 11), GS (4 + 4) 8 (n = 7) on TMA slides containing 2 to 3 cores per sample. Benign adjacent prostate tissue was present in 40 of the 51 cases. Tissue was collected in the Department of Pathology at the Cleveland Clinic as part of an IRB-approved protocol.

### Immunohistochemical staining for p62 in human prostate cancer samples

Immunohistochemistry with a monoclonal p62 antibody was performed on formalin-fixed paraffin-embedded TMA sections mounted on poly L-lysine-coated slides. The sections were deparaffinized in xylene and rehydrated through graded alcohols into distilled water. Antigen retrieval was in 0.1 M citrate buffer using a pressure cooker at 95°C for 15 min. Immunostaining was performed by using an immunohistochemistry kit according to the manufacturer’s instructions (Invitrogen) after incubation with primary antibody to p62 (Santa Cruz Biotechnologies) for 1 h. Non-immune pooled mouse immunoglobulin was used as a negative control. Sections were incubated in secondary antibody for 1 h followed by colorimetric detection using chromogen in accordance with the manufacturers’ protocols (Dako). Slides were then counterstained with haematoxylin, rinsed, and dehydrated through graded alcohols into non-aqueous solution and cover-slipped with mounting media.

### Analysis of GFP-mCherry-LC3 puncta

During autophagy, LC3B-II is recruited to the autophagosomal membranes and continues to be present on the membranes of completed autophagosomes, which can be visualized as a yellow signal because GFP and mCherry co-localize. In autolysosomes, because of the acidic pH, the GFP fluorescence is diminished while mCherry still remains stable. Thus, the conversion of yellow LC3B-II puncta to red LC3-II puncta provides a readout for autophagic flux. Cells with stable expression of GFP-mCherry-LC3 were grown overnight before treatment and fixed with 2% paraformaldehyde, washed several times with PBS, mounted using Vectashield, and analyzed using an HCX Plan Apo 63×/1.4N.A. oil immersion objective lens on a Leica TCS-SP2 confocal microscope (Leica Microsystems AG). LC3B puncta were quantified using the Red and Green Puncta Co-localization Macro with the Image J program, as described [29,30].

### Confocal immunostaining

Cells were plated at 2 × 10^5^ cells/cm^2^ on 22 × 22 mm coverslips in 35-mm culture dishes. Immunostaining was performed as previously described [30]. Briefly, following the respective treatments, cells were fixed with 2.0% paraformaldehyde/PBS for 15 min, washed 3× for 10 min each, permeabilized with 0.1% Triton X-100 in PBS for 5 min and blocked in 10% FBS in PBS for 1 h. The coverslips were then immunostained using the antibodies diluted in blocking buffer, followed by fluorescently-conjugated secondary antibody. DAPI was added to stain nuclei before the penultimate washing. They were then mounted in Vectashield (Vector Laboratories). Images were collected using an HCX Plan Apo 63×/1.4N.A. oil immersion objective lens on a Leica TCS-SP2 confocal microscope (Leica Microsystems AG).

### Electron microscopic analyses

Cells were immediately fixed in 2.5% glutaraldehyde/4% formaldehyde in 0.1 M Cacodylate buffer, pH 7.3, for 24 h, followed by post-fixation with 1% osmium tetroxide for 1 h. After *en bloc* staining and dehydration with ethanol, the samples were embedded with eponate 12 medium (Ted Pella Inc, Redding, CA). Thin sections (85 nm) were cut with a diamond knife, double-stained with uranyl acetate and lead citrate, and analyzed using a Philips CM12 electron microscope (FEI Company) operated at 60 kv. Cells with more than 10 vacuoles were scored as autophagy positive. The autophagic area was quantified as described previously [30]. At least 10 cells per sample were used for quantitation. The size of autophagic structures was represented as relative area values calculated by selecting specific areas using Image J. The autophagic area was calculated as the percentage fraction normalized to the total cytoplasmic area.

### Lentiviral transduction of shRNA

shRNA target sequences were: *ATG7* 3′UTR, 5′-CCGGGCCTGCTGAGGAGCTC TCCATCTCGAGATGGAGAGCTCCTCAGCAGGCTTTT-3′ and *LAMP2* CDS-5′-CCGGGCC ATCAGAATTCCAT TGAATCTCGAGATTCAATGGA ATTCTGATGGCTTTTT-3′. 293T cells were transfected with these shRNAs together with the pVSVG and dr8.2 plasmids in a 3:1:1 ratio using the Fugene transfection reagent. Media containing viral particles was then collected 24 h after transfection, passed through a 0.8-micron filter, and added to PC3 and C4-2 cells along with polybrene (10 μg/ml). After overnight incubation at 37°C, the medium was replenished, and cells were selected for puromycin resistance (2 μg/ml) for 3 days, after which knockdown was further validated. For control pLKO.1 vector and non-target shRNA-expressing stable cell lines were generated as described [30].

### Cell death and survival analyses

Cell viability was determined by staining with annexinV-FITC and propidium iodide (PI) followed by flow cytometric analysis on a FACScan with a 488 nm argon laser (BD Biosciences). Analyses were performed with the Cell Quest program. For clonogenic survival, cells were plated in 6-well plates in triplicate. Following drug treatments, cells were allowed to grow for 14 days, fixed, and stained in methanol:acetic acid (75:25, v/v) containing 0.5% crystal violet (w/v) to visualize colonies of at least 50 cells. The absolute number of colonies was plotted.

### Confocal time-lapse imaging

Cells were grown in 35-mm glass bottom dishes (MatTek) and imaged using an UltraVIEW VoX spinning disc confocal microscope (Perkin Elmer) equipped with a high-sensitivity cooled 14-bit EMCCD C9100-13 camera (Hamamatsu). During imaging, cells were kept in a heated incubation chamber at 37°C with CO_2_ (LiveCell, Pathology Devices, Inc.). Volocity image acquisition software was used to capture the images (Perkin Elmer). The track analysis and intensity measurements were done with Image Pro 7.0 (Media Cybernetics).

### Statistical analyses

We analyzed the correlation between cytoplasmic expression of p62 and Gleason score by Pearson’s correlation analysis (Prism v.5, GraphPad Software, Inc.). All the remaining data were obtained from at least three independent experiments carried out in triplicate with the error bars denoting SEM. *P* values were determined by Student’s *t*-test (Microsoft Excel).

## Abbreviations

Apo2L/TRAIL: Apo2 ligand/tumor necrosis factor-related apoptosis-inducing ligand; BECN1: Beclin-1; CQ: Chloroquine; LC3B: Microtubule-associated protein 1 light chain 3 beta; p62: p62/SQSTM1; PCa: Prostate cancer; 3-MA: 3-methyladenine; TMA: Tissue microarray; TR: Apo2L/TRAIL treatment.

## Competing interests

The authors declare that they have no competing interests.

## Authors’ contributions

Conceived and designed the experiments: KS, AA. Performed the experiments: KS, AS, CMM. Contributed clinical samples and expertise: CMG, CMM, DH, EAK. Analyzed the data: KS, AS, BPR, CMM. Contributed reagents/materials/analysis tools: JAD, WDH. Wrote the paper: KS, AS, AA. All authors read and approved the final manuscript.

## Supplementary Material

Additional file 1: Movie S1 GFP-mCherry-LC3 puncta formation in PC3 cells. Confocal time-lapse video of PC3 cells stably expressing GFP-mCherry-LC3 following TR. The arrow represents one of the highlighted autophagosomes shown in Figure 3A. Click here for file

Additional file 2: Movie S2 GFP-mCherry-LC3 puncta formation in C4-2 cells. Confocal time-lapse video of C4-2 cells stably expressing GFP-mCherry-LC3 following TR. The arrow represents one of the highlighted autophagosomes shown in Figure 3A. Click here for file

Additional file 3: Figure S1 Autophagic flux is higher in TR-resistant cells. PC3 and C4-2 cells were treated with Apo2L/TRAIL (TR) ± chloroquine (CQ). (A) Representative confocal immunostaining of endogenous LC3 puncta-positive cells. (*blue= DAPI, red=LC3*). (B) Western blotting analysis of p62 and β-actin was used as loading control. (C) Cell death is shown as percentage of cells with sub-G1 DNA content at the indicated time following TR (*p<0.001). ). Indicated cell lines were treated with TR ± CQ and p62 levels were analyzed by (D) Western blotting and β-actin was used as a loading control. (E) confocal immunostaining of endogenous p62. Click here for file

Additional file 4: Figure S2 Inhibition of autophagy in PC3 and C4-2 cells by shRNA-mediated stable knockdown of ATG7 and LAMP2. (A and B) Cells were treated with HBSS in order to determine starvation-induced autuohagy. Western blot analysis showing levels of LC3, p62, ATG7 and LAMP2 in PC3 and C4-2 cells stably expressing shATG7 or shLAMP2 (+), respectively, compared to non-target shRNA controls (NT) (-). β-actin served as a loading control. Click here for file

Additional file 5: Figure S3 Impaired autophagic degradation, led to caspase-8 activation and apoptosis in TR-sensitive cells. (A) Annexin V-FITC/PI staining to determine % cell death in PC3 and C4-2 cells stably- expressing non-target shRNA, shATG7, or shLAMP2, at 24 h following TR (*P<0.001). (B) Annexin V-FITC/PI staining to determine % cell death in PC3 and C4-2 cells at 24 h following TR ± z-VAD or z-IETD-fmk (caspase 8 inhibitor) (*P<0.001). Click here for file
