# Supplementary figures and images for: Autophagic flux determines cell death and survival in response to Apo2L/TRAIL (dulanermin)
Source: Mol Cancer. 2014 Mar 23;13:70. doi: 10.1186/1476-4598-13-70 (PMC3998041; doi:10.1186/1476-4598-13-70)

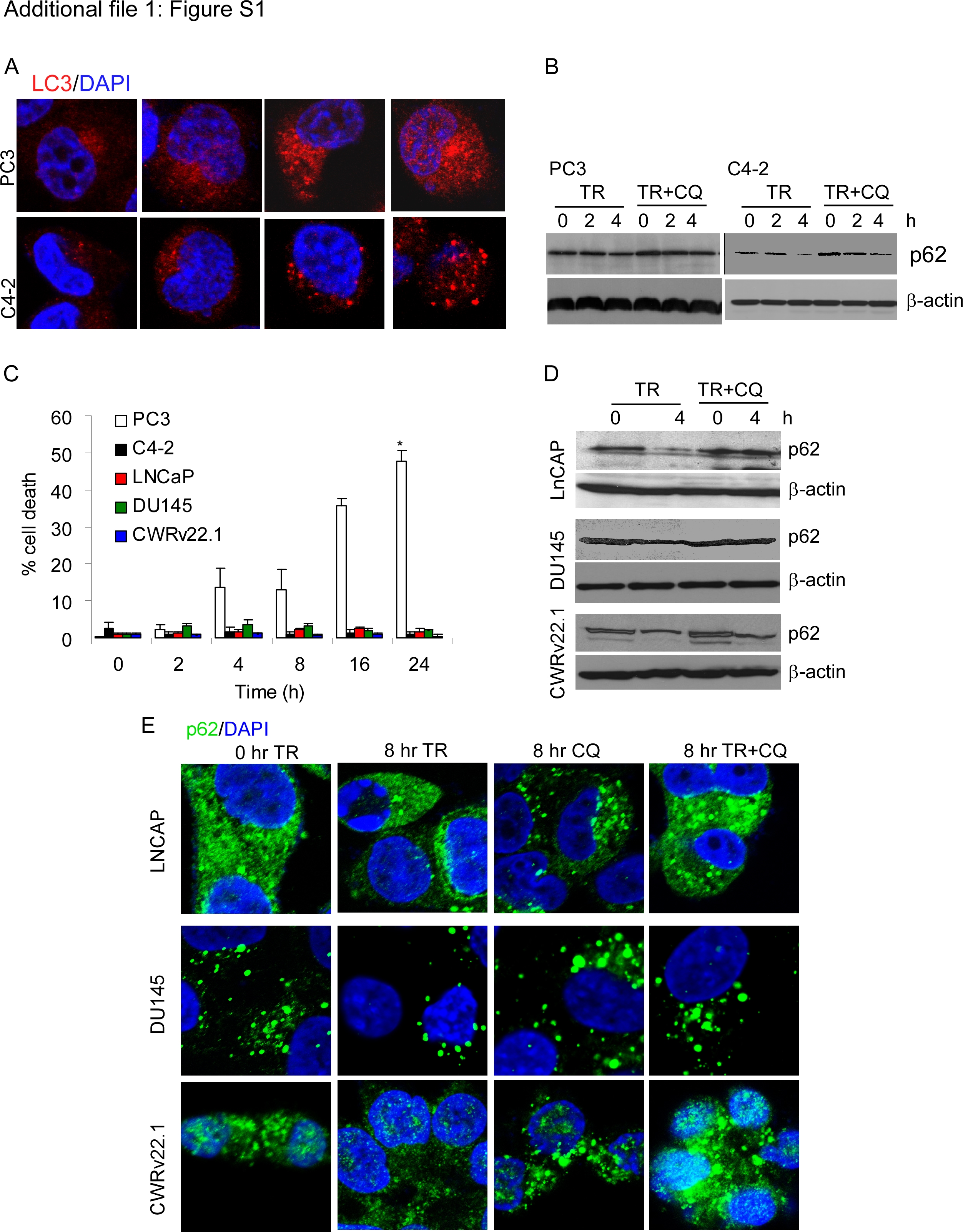

Supplement: Additional file 3: Figure S1 — Autophagic flux is higher in TR-resistant cells. PC3 and C4-2 cells were treated with Apo2L/TRAIL (TR) ± chloroquine (CQ). (A) Representative confocal immunostaining of endogenous LC3 puncta-positive cells. (blue= DAPI, red=LC3). (B) Western blotting analysis of p62 and β-actin was used as loading control. (C) Cell death is shown as percentage of cells with sub-G1 DNA content at the indicated time following TR (*p<0.001). ). Indicated cell lines were treated with TR ± CQ and p62 levels were analyzed by (D) Western blotting and β-actin was used as a loading control. (E) confocal immunostaining of endogenous p62. [file 1476-4598-13-70-S3.jpeg]

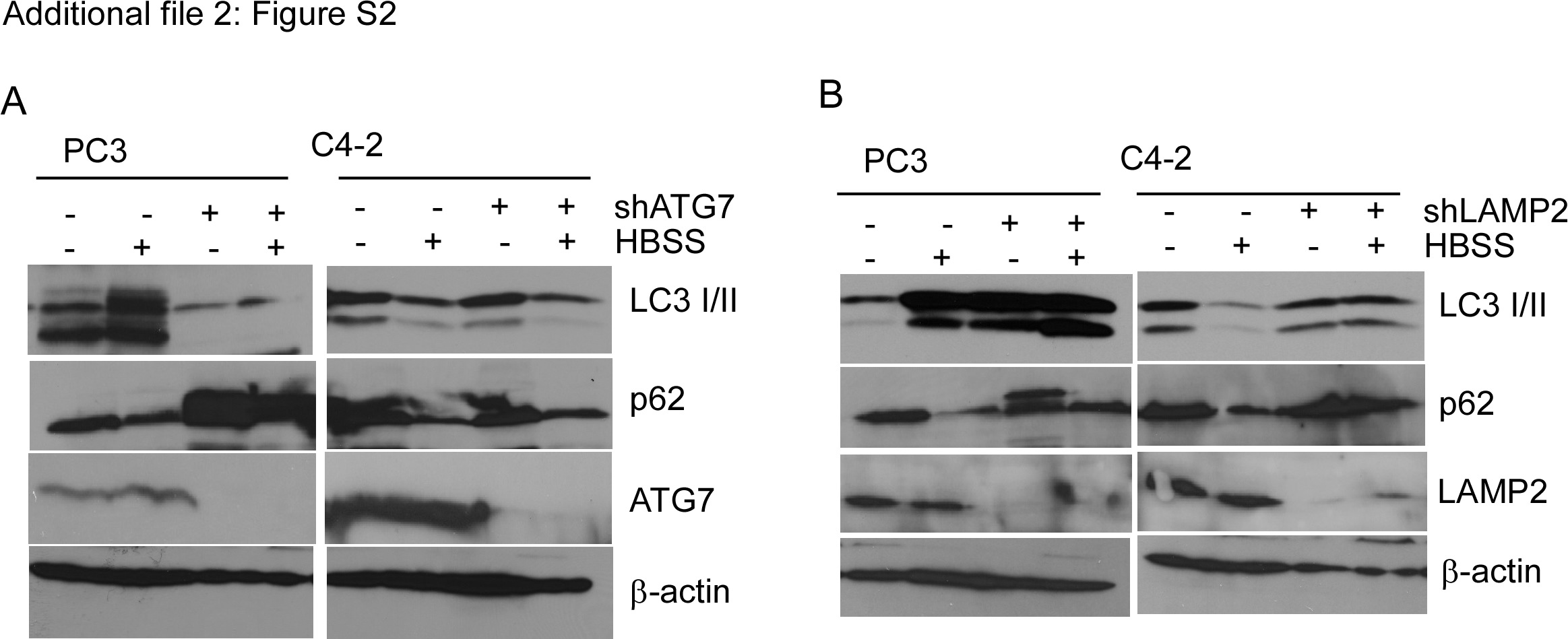

Supplement: Additional file 4: Figure S2 — Inhibition of autophagy in PC3 and C4-2 cells by shRNA-mediated stable knockdown of ATG7 and LAMP2. (A and B) Cells were treated with HBSS in order to determine starvation-induced autuohagy. Western blot analysis showing levels of LC3, p62, ATG7 and LAMP2 in PC3 and C4-2 cells stably expressing shATG7 or shLAMP2 (+), respectively, compared to non-target shRNA controls (NT) (-). β-actin served as a loading control. [file 1476-4598-13-70-S4.jpeg]

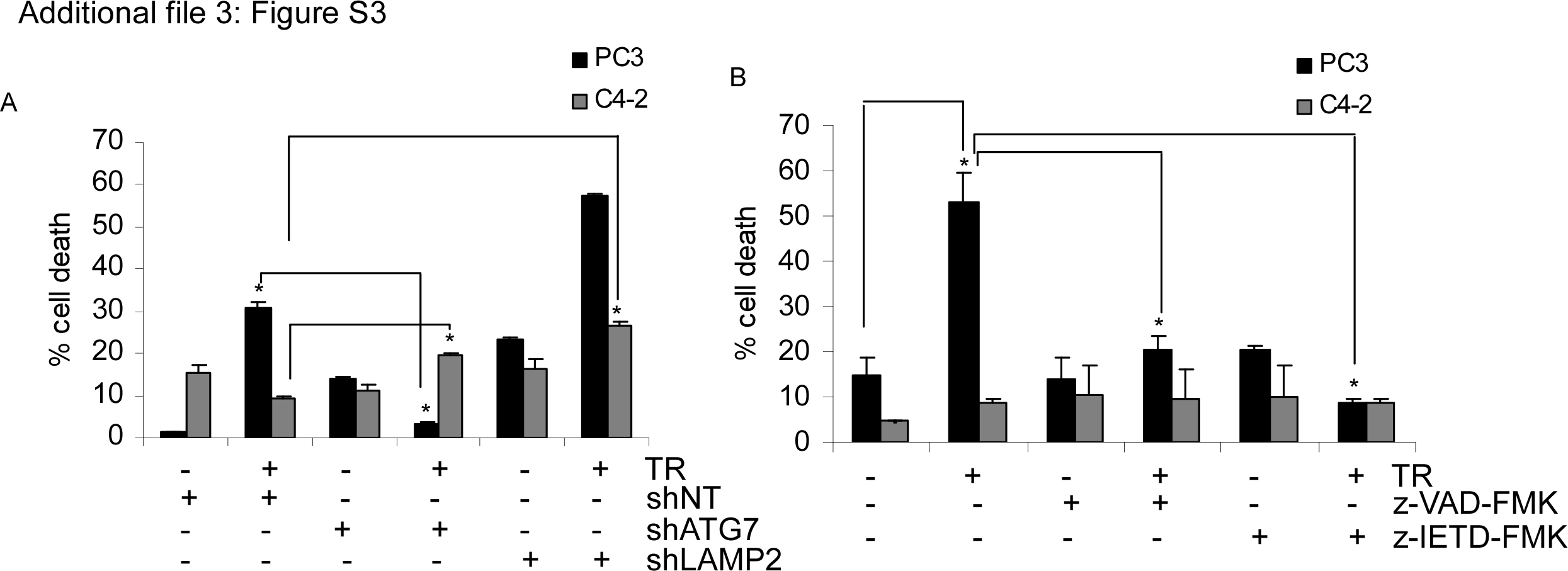

Supplement: Additional file 5: Figure S3 — Impaired autophagic degradation, led to caspase-8 activation and apoptosis in TR-sensitive cells. (A) Annexin V-FITC/PI staining to determine % cell death in PC3 and C4-2 cells stably- expressing non-target shRNA, shATG7, or shLAMP2, at 24 h following TR (*P<0.001). (B) Annexin V-FITC/PI staining to determine % cell death in PC3 and C4-2 cells at 24 h following TR ± z-VAD or z-IETD-fmk (caspase 8 inhibitor) (*P<0.001). [file 1476-4598-13-70-S5.jpeg]
